# Supplementary material for: Hidden non-collinear spin-order induced topological surface states
Source: Nat Commun. 2024 Apr 5;15:2937. doi: 10.1038/s41467-024-47340-2 (PMC10997621; doi:10.1038/s41467-024-47340-2)
Supplement: Supplementary file 1 — Supplementary information [file 41467_2024_47340_MOESM1_ESM.pdf]

Supplementary Information for

Hidden non-collinear spin-order induced topological surface states

Zengle Huang,[1†] Hemian Yi,[2†] Daniel Kaplan,[1,3†] Lujin Min,[2] Hengxin Tan,[3] Ying-ting Chan,[1] Zhiqiang Mao,[2] Binghai Yan,[3] Cui-Zu Chang,[2] Weida Wu[1*]

[1]Department of Physics & Astronomy, Rutgers University, Piscataway, New Jersey 08854, USA
[2]Department of Physics, The Pennsylvania State University, University Park, Pennsylvania 16802, USA
[3]Department of Condensed Matter Physics, Weizmann Institute of Science, Rehovot, Israel

[*]To whom correspondence should be addressed; E-mail: wdwu@physics.rutgers.edu.
[†]These authors contributed equally to this work

March 12, 2024

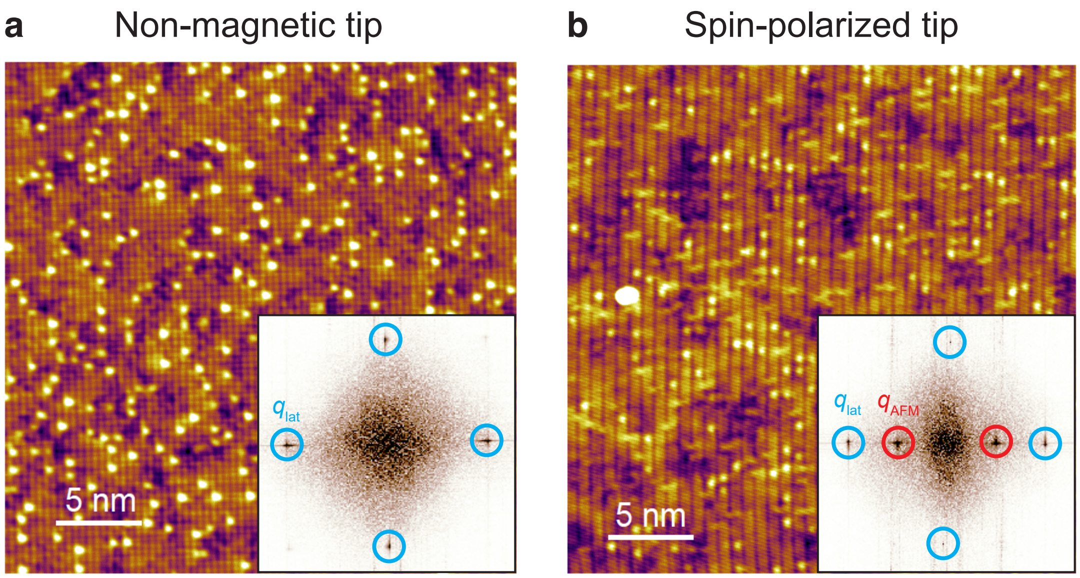

Supplementary Fig. 1: **Preparation and demonstration of spin-polarized STM tip on FeTe.** **a**, A 30×30 nm^2 topographic image of FeTe taken with a non-magnetic tip. V_{bias}=+60 mV, I=1 nA. The inset is the FFT. Only two pairs of lattice Bragg peaks are present. **b**, A 30×30 nm^2 topographic image of FeTe taken with a spin-polarized tip. V_{bias}=+100 mV, I=1 nA. The inset is the FFT. There is an additional pair of peaks corresponding to the bi-collinear AFM order.

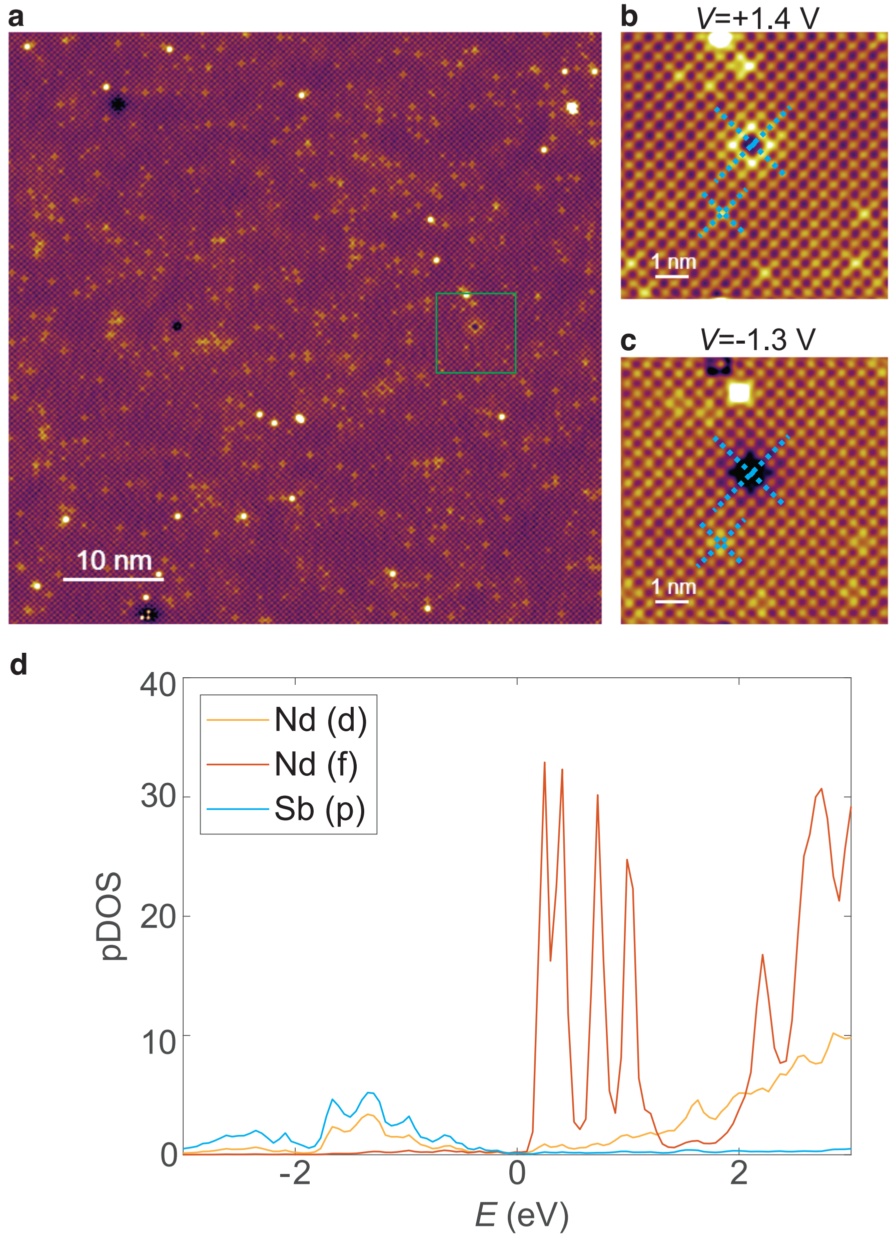

Supplementary Fig. 2: **Large-area and bias-dependent topographic image of NdSb.** **a**, A 60×60 nm^2 topographic image of NdSb. V_{bias}=+1.4 V, I=1.2 nA. **b**, **c**, Zoom-in images of the green box in a. **b** is taken at V_{bias}=+1.4 V, I=1.2 nA and **c** at V_{bias}=+1.4 V, I=1.2 nA. The blue dashed lines highlight the center of defects on the surface. **d**, The orbital-decomposed density of states for Nd and Sb sites in the $2q$ configuration of NdSb. The yellow, orange and blue lines describe the density of Nd d, Nd f and Sb p orbitals, respectively.

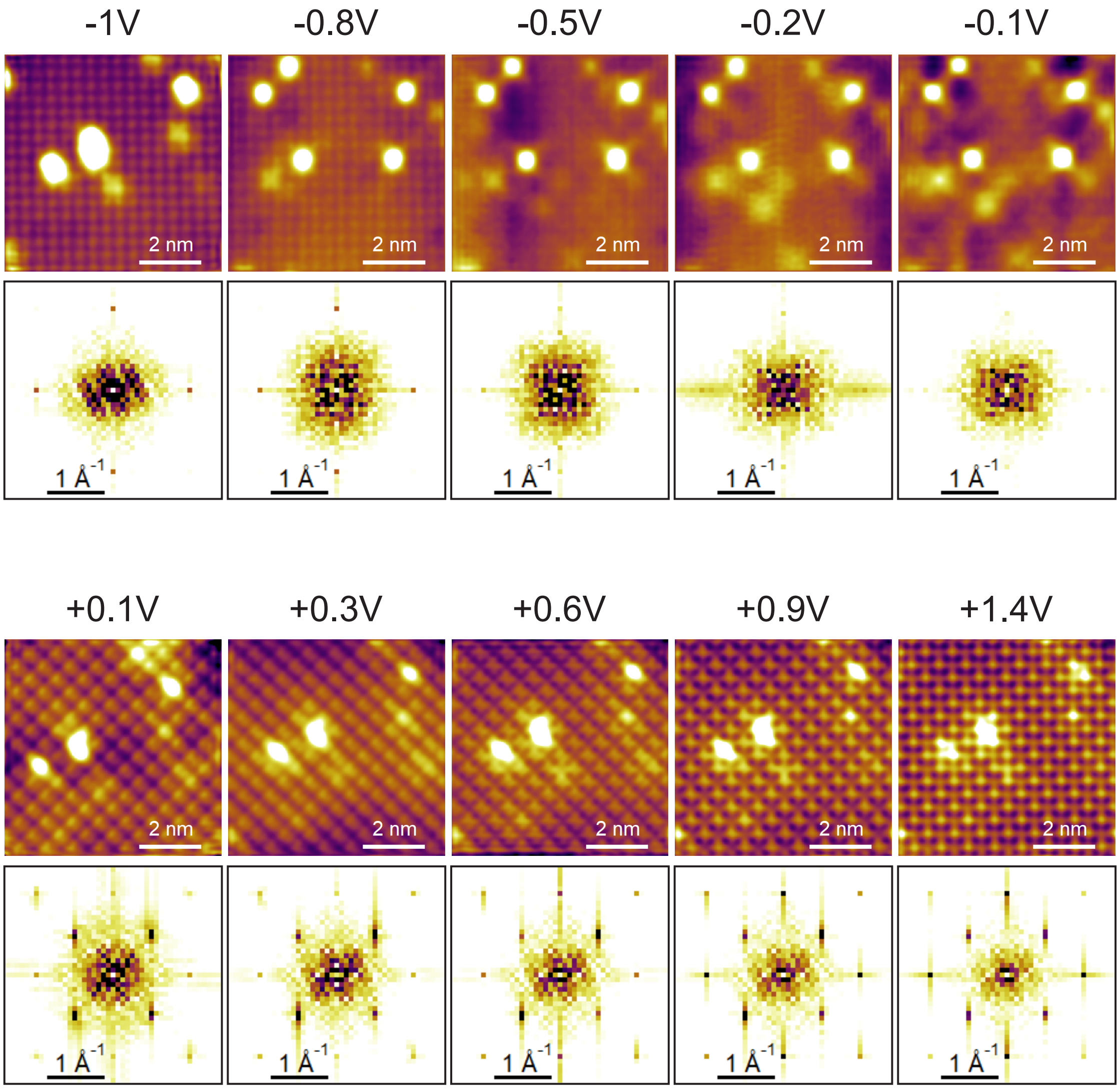

Supplementary Fig. 3: **Bias-dependent spin-polarized topographic images at 4 K.** Bias-dependent spin-polarized topographic images from −1 V to +1.4 V. The diminished magnetic contrast around −1 V indicates that at large negative bias, the atom-like features are nonmagnetic Sb atoms, while at positive bias the atom-like features are magnetic Nd atoms.

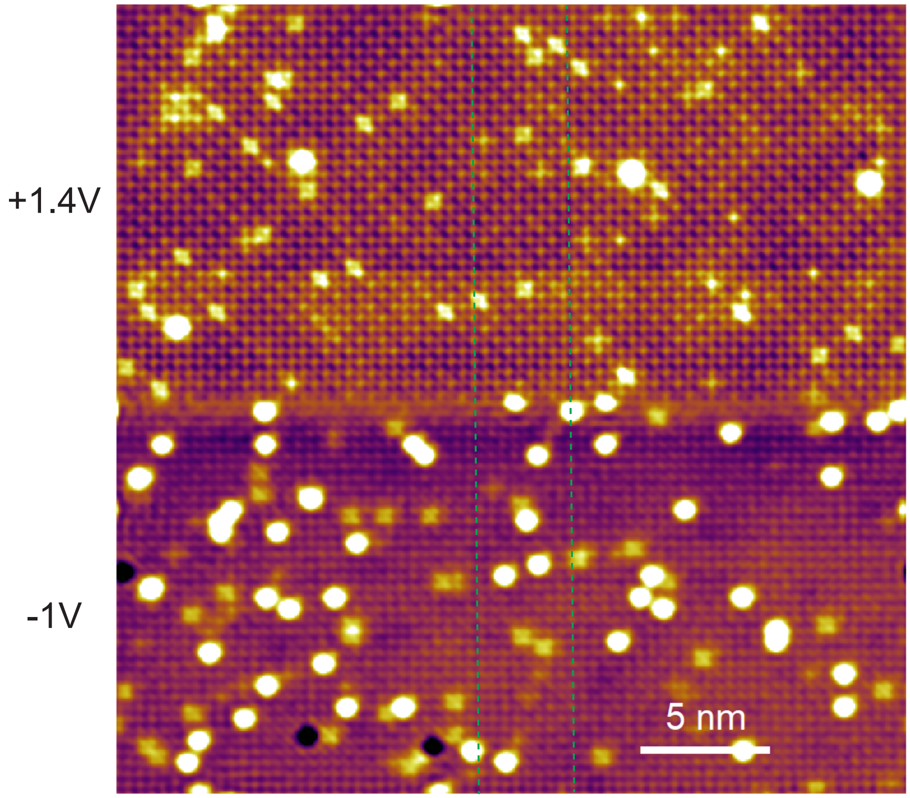

Supplementary Fig. 4: **Comparison between spin-polarized topographic images at +1.4 V and −1 V.** A 30×30 nm^2 spin-polarized topographic image whose lower part is taken at −1 V and upper part +1.4 V. The green dashed lines track the lattice shift between +1.4 V and −1 V. The spin-polarized images reproduce the lattice shift observed in nonmagnetic images. Tunneling current I=1 nA.

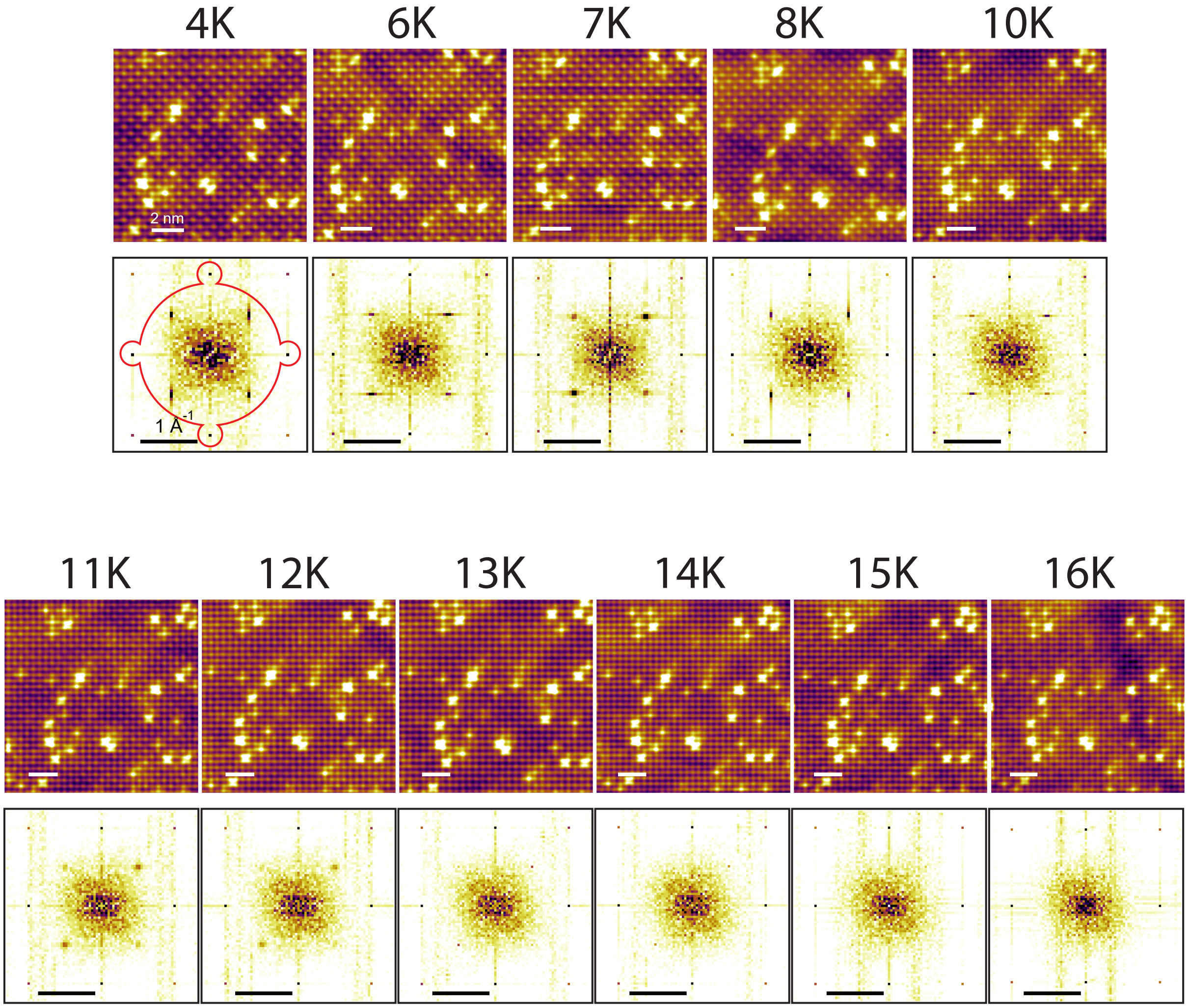

Supplementary Fig. 5: **Complete set of temperature-dependent spin-polarized topographic images used in the main text.** Temperature-dependent spin-polarized topographic images taken in the same area. V_{bias}=1.4 V, I=1.2 nA. The top row is the filtered and drift-corrected STM images and the bottom row is the FFT. The filtered STM images are obtained from the inverse FFT of the FFT within the red mask.

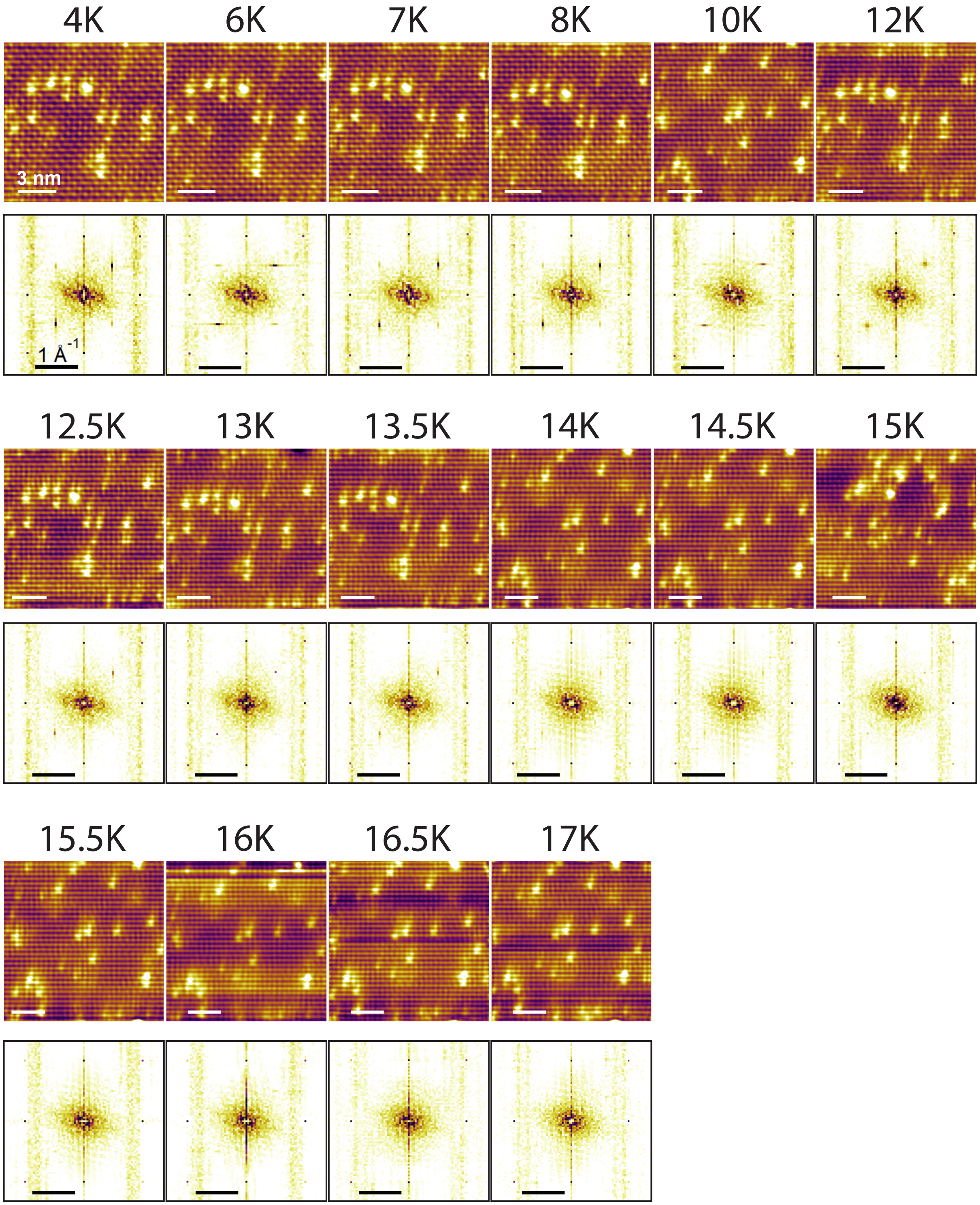

Supplementary Fig. 6: **Temperature-dependent spin-polarized images dataset #2.** Temperature-dependent spin-polarized STM images dataset #2 obtained with a magnetic tip prepared separately and another piece of NdSb sample than that in Fig.5 and the main text. Tunneling condition: V_{bias}=+1.4 V, I=1.2 nA.

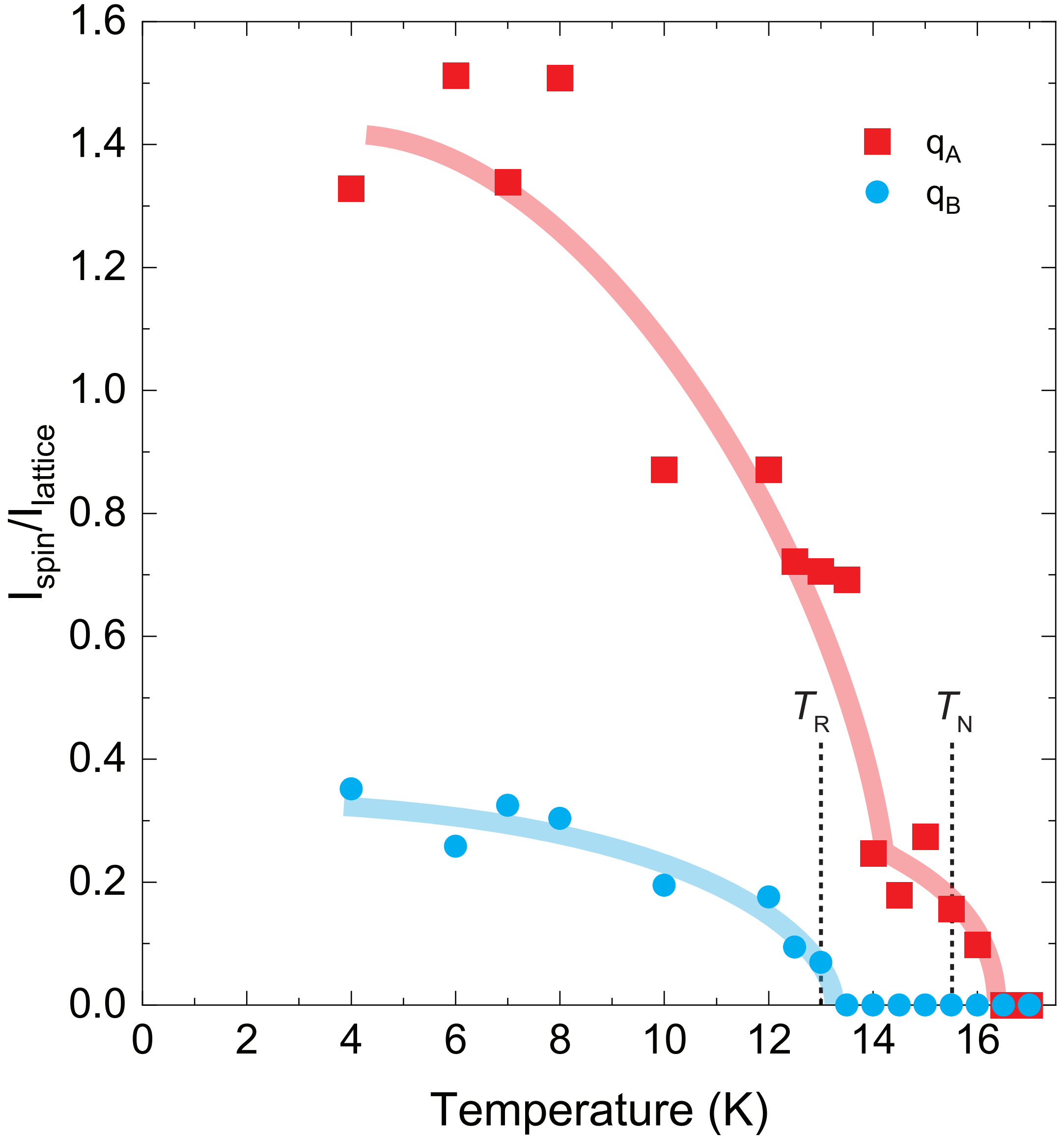

Supplementary Fig. 7: **Temperature evolution of normalized magnetic peak intensity obtained from dataset #2 in Fig. 6.**

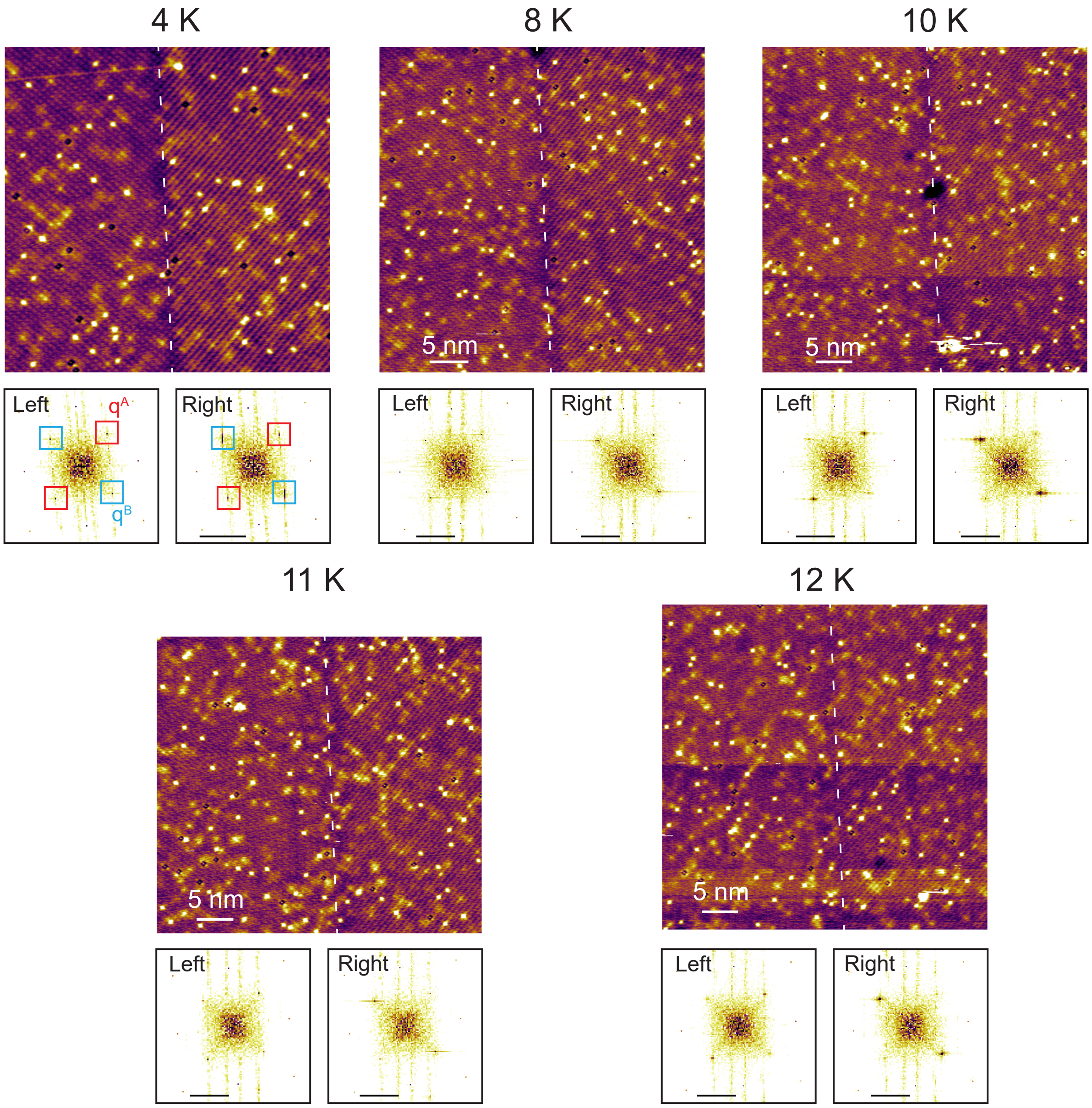

Supplementary Fig. 8: **Temperature evolution of adjacent magnetic domains.** Tunneling condition: V_{bias}=+0.3 V, I=0.5 nA

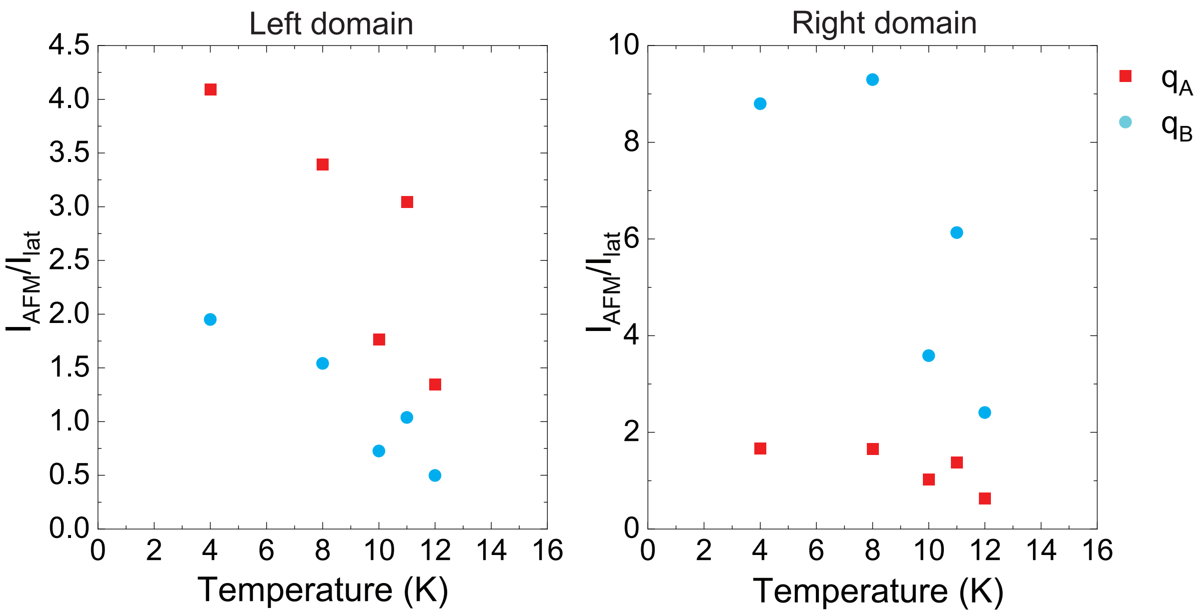

Supplementary Fig. 9: **Temperature evolution of normalized magnetic peak intensities of adjacent magnetic domains.** The intensities are obtained from Supplementary Fig. 8. The magnetic peak intensities in both domains decay as the temperature rises.

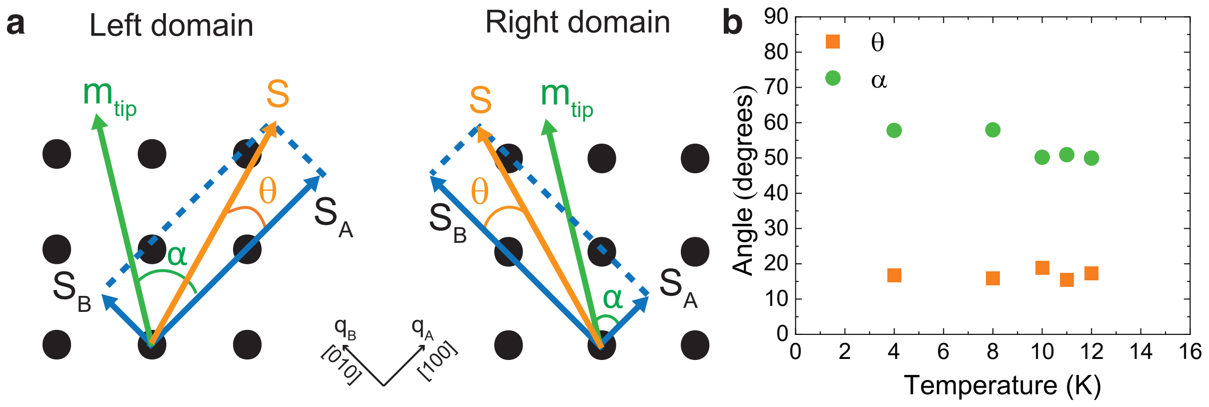

Supplementary Fig. 10: **Estimation of Nd spin moment angle. a**, Schematics of tip spin moment m_{tip} and the Nd spin moment S on the Nd lattice (black dots). S_A and S_B are the projection of Nd spin onto q_A and q_B, respectively; α is the angle between the tip moment and q_A-axis, or [100]; θ is the angle between Nd spin moment and q_A-axis. **b**, The estimated tip moment (α) and Nd spin moment (θ) angles relative to the q_A-axis.

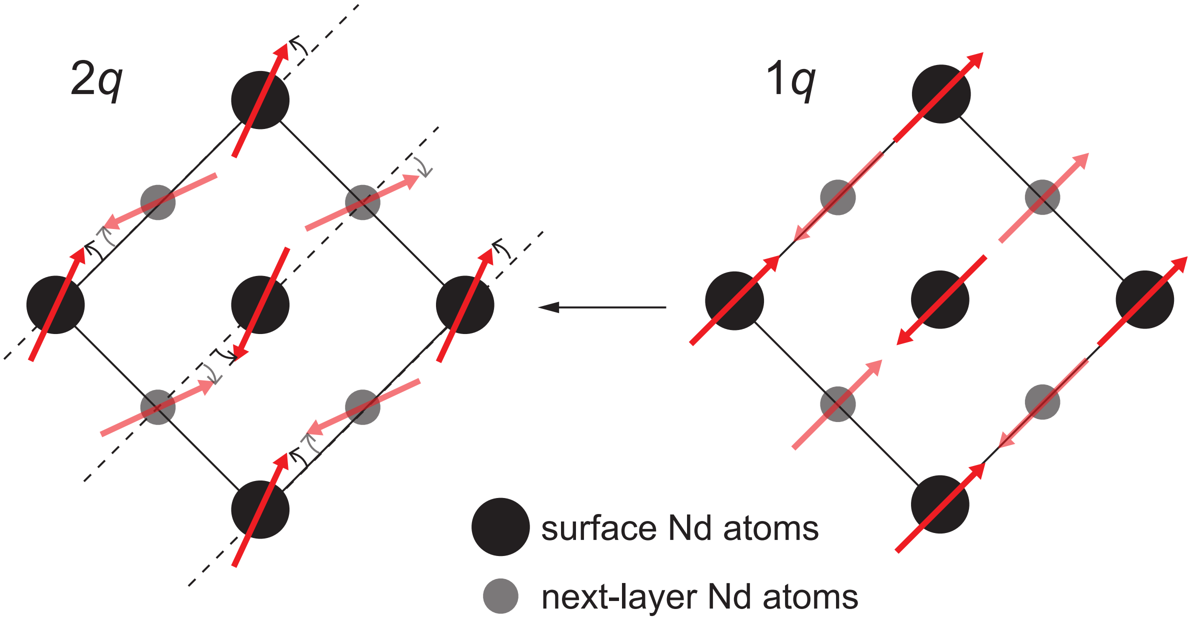

Supplementary Fig. 11: **Schematics of Nd spin moments rotation below T_R.** The Nd spin moments on the neighboring layers rotate in opposite directions, transforming from the collinear $1q$ to non-collinear $2q$ magnetic order.

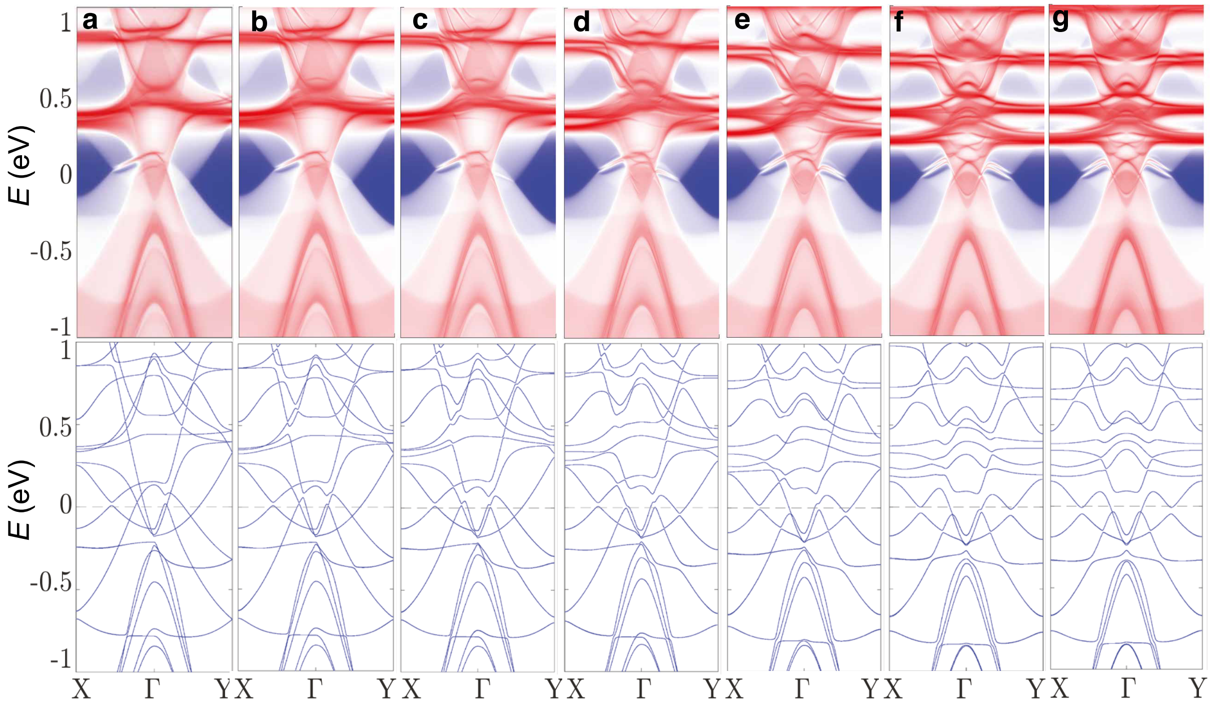

Supplementary Fig. 12: **Evolution of surface states, bulk bands and magnetic order in NdSb. a-g**, surface states (top) and bulk band structures along the $X - \Gamma - Y$ line with the magnetic moment angle relative to the x-axis increasing as $0, 5, 10, 20, 30, 40, 45$ degrees, respectively. **a** corresponds to the $1q$ configuration. **g** corresponds to 2q with magnetic moments along the diagonal in the (001) plane. The experimental determination of the magnetic moment puts the angle close to 20 degrees, represented by panel (**d**).

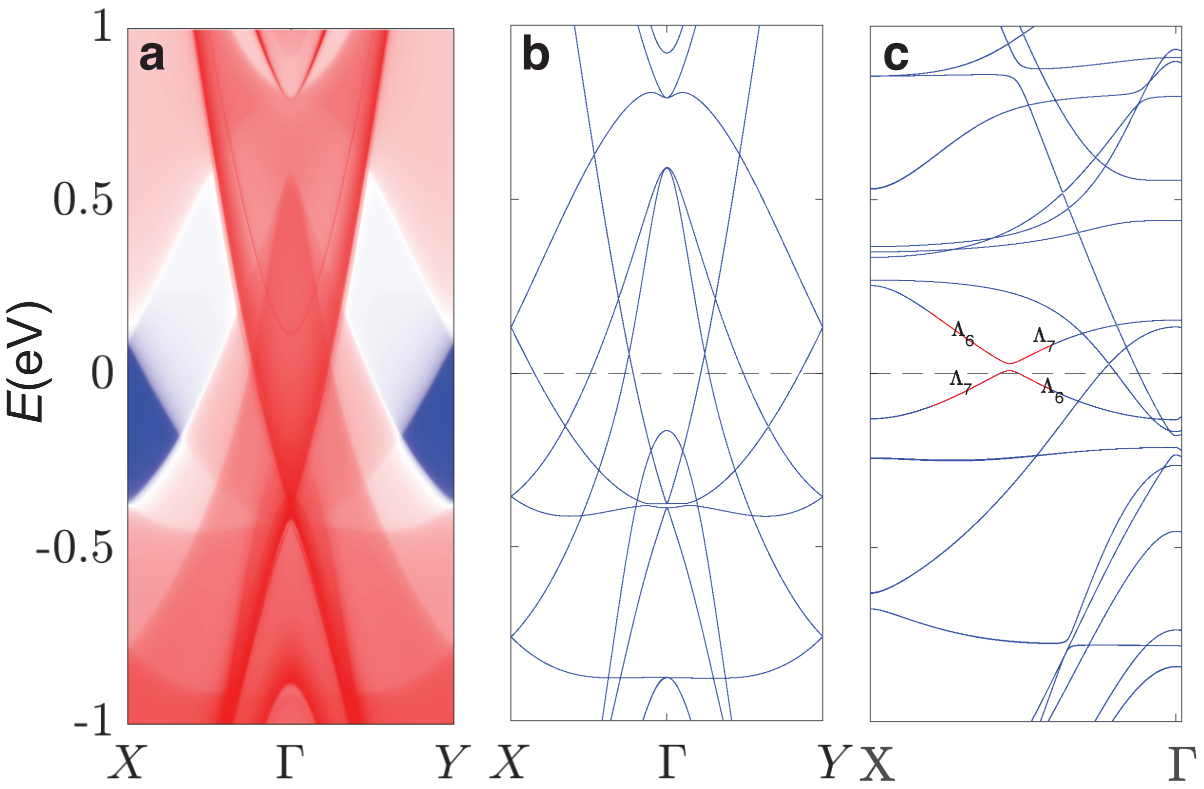

Supplementary Fig. 13: **Paramagnetic surface states and bulk bands, and demonstration of the band inversion for $\theta = 0$ (1q) along $\Gamma - X$.** **a**, Surface bands along $X - \Gamma - Y$ for the paramagnetic phase for the conventional cubic cell. Note the absence of any surface states. **b**, Bulk bands along the $X - \Gamma - Y$ path showing trivial crossing due to folding within the conventional cell. **a** and **b** do not contain any f electron contribution, as these are kept frozen in the core for purposes of accurately capturing the non-magnetic ground state. **c**, Bulk band inversion for $\theta = 0$, 1q state. The highlighted bands (in red) correspond to the position in momentum space where bands anti-cross. The irreducible representations of C_{4x} are superimposed next to the bands, showing the topological character of the gap.

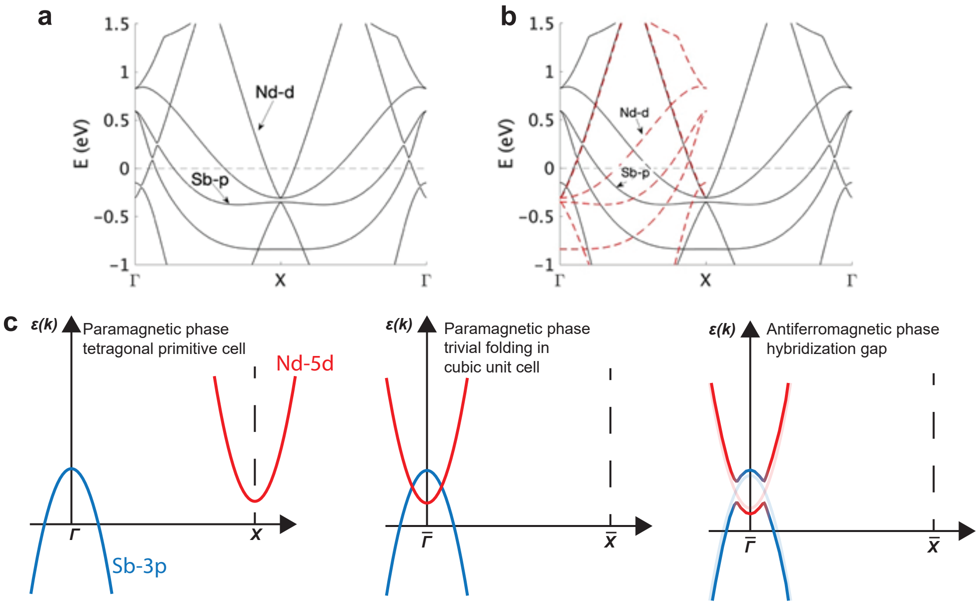

Supplementary Fig. 14: **Illustration of band folding and hybridization gap opening.** **a**, A schematic of the bulk bands in the paramagnetic phase of NdSb. **b**, A schematic of bulk bands in the PM phase of NdSb when the bands are trivially folded. Note that crossings are now enabled, between Nd-d and Sb-p orbitals. The lack of magnetic order prevents a gap opening (due to the higher cubic symmetry present). c) A simplified cartoon illustrating the band folding and hybridization gap opening. In the paramagnetic phase, SOC causes the conduction band minimum at the X point to be lowered than the valence band maximum at the Γ point (as in (**a**)). Below Neel temperature, When magnetic order is created and the symmetry is reduced, the conduction band is folded to the Γ point and overlaps with the valence band, causing the band antic-crossings and opening a topological band gap.
